# Deciphering the Functional Role of RIPK4 in Melanoma

**DOI:** 10.3390/ijms222111504

**Published:** 2021-10-25

**Authors:** Ewelina Madej, Damian Ryszawy, Anna A. Brożyna, Malgorzata Czyz, Jaroslaw Czyz, Agnieszka Wolnicka-Glubisz

**Affiliations:** 1 Faculty of Biochemistry, Biophysics and Biotechnology, Department of Biophysics, Jagiellonian University, 7 Gronostajowa Street, 30-387 Krakow, Poland; ewel.madej@doctoral.uj.edu.pl; 2 Faculty of Biochemistry, Biophysics and Biotechnology, Department of Cell Biology, Jagiellonian University, 7 Gronostajowa Street, 30-387 Krakow, Poland; damian.ryszawy@uj.edu (D.R.); jarek.czyz@uj.edu.pl (J.C.); 3 Faculty of Biological and Veterinary Sciences, Institute of Biology, Department of Human Biology, Nicolaus Copernicus University, 1 Lwowska Street, 87-100 Torun, Poland; anna.brozyna@umk.pl; 4 Department of Molecular Biology of Cancer Lodz, Medical University of Lodz, 6/8 Mazowiecka Street, 92-215 Lodz, Poland; malgorzata.czyz@umed.lodz.pl

**Keywords:** invasive potential, melanoma, MMPs, NF-κB, RIPK4

## Abstract

The receptor-interacting protein kinase 4 (RIPK4) plays an important role in the development and maintenance of various tissues including skin, but its role in melanoma has not been reported. Using patient-derived cell lines and clinical samples, we show that RIPK4 is expressed in melanomas at different levels. This heterogenous expression, together with very low level of RIPK4 in melanocytes, indicates that the role of this kinase in melanoma is context-dependent. While the analysis of microarray data has revealed no straightforward correlation between the stage of melanoma progression and RIPK4 expression in vivo, relatively high levels of RIPK4 are in metastatic melanoma cell lines. RIPK4 down-regulation by siRNA resulted in the attenuation of invasive potential as assessed by time-lapse video microscopy, wound-healing and transmigration assays. These effects were accompanied by reduced level of pro-invasive proteins such as MMP9, MMP2, and N-cadherin. Incubation of melanoma cells with phorbol ester (PMA) increased PKC-1β level and hyperphosphorylation of RIPK4 resulting in degradation of RIPK4. Interestingly, incubation of cells with PMA for short and long durations revealed that cell migration is controlled by the NF-κB signaling in a RIPK4-dependent (RIPK4^high^) or independent (RIPK4^low^) manner depending on cell origin (distant or lymph node metastasis) or phenotype (mesenchymal or epithelial).

## 1. Introduction

The receptor-interacting serine/threonine kinase protein 4 (RIPK4, also known as DIK or PKK) is a highly conserved member of the RIP family of serine-threonine kinases containing an N-terminal RIP-like kinase domain and a C-terminal region characterized by 11 ankyrin repeats [1,2]. In humans, RIPK4 is widely expressed in numerous tissues [1,3], and two isoforms of 86 and 92 kDa are found as the result of alternative splicing. Mutations in RIPK4, including loss-of-function mutation, have been detected in keratinocytes of patients with Bartsocas-Papas syndrome [4,5]. Deletion of RIPK4 in mice leads to death in the first hours of postnatal life. Interestingly, the observed deformations of RIPK4 KO mice are phenotypically similar to IKK (I kappa B kinase) KO mice, suggesting that RIPK4 is involved in NF-κB-dependent keratinocyte differentiation [6,7]. These findings are in contrast to conflicting data on the RIPK4 role in cancer [7]. Depending on the context, RIPK4 acts as a cancer “suppressor” or oncogene [8,9,10,11,12,13]. While RIPK4 down-regulation was observed during the development of neoplasms of liver [8], tongue [9], and lung [10], high RIPK4 levels are correlated with progression and poor prognosis in cervical squamous cell carcinoma [11], lymph node metastasis of cervical cancer [12], and increased invasiveness of pancreatic cancer [13]. Despite the role of RIPK4 in skin homeostasis [7], its function in melanoma progression has not yet been investigated.

Melanoma that arises from transformed melanocytes represents the most lethal type of skin tumor. Although a decreasing trend in fatal melanoma cases is observed in the United States and the majority of European countries [14,15], as a result of early detection and the clinical use of the BRAF^V600^/MEK inhibitors and immune checkpoint inhibitors for advanced stage melanomas [16,17], the incidence and mortality rates are still unacceptably high [14,18]. Numerous questions about melanoma diagnosis, development of metastasis, lack of response and resistance to immune- and targeted- therapy still need to be addressed [18]. Melanoma initiation, progression, and resistance to treatment are determined by various signaling pathways, including RAF/MEK/ERK [19], PKC/NF-κB [20,21,22,23], and Wnt/β-catenin signaling [24]. Notably, these pathways have also been identified as targets of RIPK4 [25,26,27].

We investigated interactions between RIPK4, PKC and NF-κB pathways in melanoma and the role of RIPK4 in regulating the invasive potential of melanoma cells. To this end, we used transcriptomic analyses of RIPK4 expression levels in melanoma specimens from clinical trials, IHC in melanoma biopsies, and protein level in patient-derived and commercially available melanoma cell lines. The results indicate that RIPK4 contributes to melanoma invasive potential in a lineage-specific/NF-κB-dependent manner.

## 2. Results

### 2.1. RIPK4 Is Heterogeneously Expressed in Melanoma Cells

**Figure 1:**
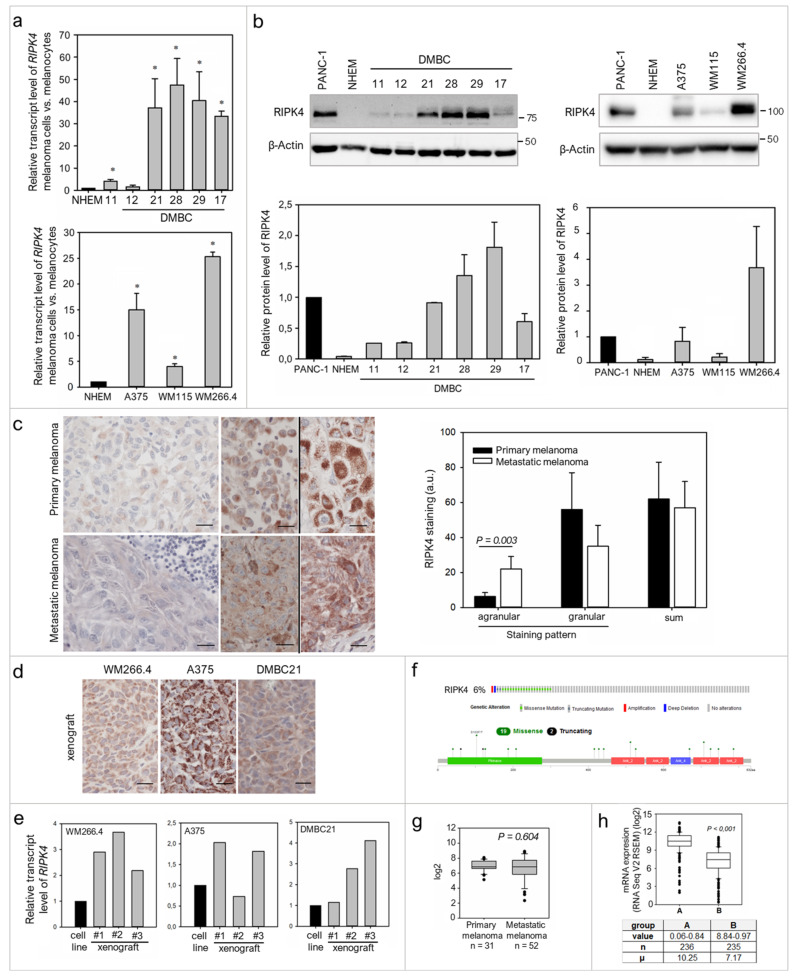
Diverse expression of RIPK4 in melanoma. (**a**) RIPK4 RNA expression level presented as fold change in comparison to melanocytes (NHEM) where expression was set as 1. mRNA expression in patient-derived melanoma cell lines DMBC 11, 12, 17, 21, 28, 29 was normalized to RPS17 (upper) and in established melanoma cell lines: A375, WM115, WM266.4 to GAPDH (lower). *n* = 3. * *p* < 0.05. (**b**) RIPK4 protein levels were assessed by Western blotting with densitometry. A human pancreatic cancer cell line, PANC-1, was used as a positive control. *n* = 3. (**c**) Representative IHC images for RIPK4 in primary and metastatic melanomas from the same patients, either with low (left; agranular staining pattern) or high (right; two samples separated with black line; agranular and granular staining pattern) staining intensity. Scale bar: 20 µm. Bar graph of differences between primary and metastatic melanomas measured Student’s *t*-test with Wilcoxon matched-pairs signed ranked for paired data from the same patients. *n* = 7. (**d**) Representative IHC images of xenografts from WM266.4, A375 and DMBC21 cells in NOD/SCID mice. Scale bar: 20 µm. (**e**) RIPK4 mRNA in xenografts compared to parental cell line and normalized to GAPDH. *n* = 3. (**f**) The frequency of RIPK4 mutations and truncations in melanoma (6%) and distribution of RIPK4 mutations in melanoma across protein domains. (**g**) RIPK4 mRNA expression in metastatic vs. primary melanoma according to dataset from GEO database, GDS 3966. (**h**) Correlation between methylation of RIPK4 gene and RIPK4 mRNA expression. A and B are groups from median of RIPK4: methylation (HM450), *n*—the number of samples, μ—Mean log2 expression of the listed gene in samples.

To date, there is no evidence that RIPK4 plays any role in melanoma, thus we first investigated its level in established melanoma cell lines, patient-derived melanoma cell lines and clinical samples. Expression of RIPK4 in melanoma cells at the mRNA and protein levels was higher in melanoma cells than in normal melanocytes (Figure 1a,b). Various levels of RIPK4 were detected in melanoma cells, from slightly above levels in melanocytes of DMBC11, DMBC12 and WM115 cell lines to highly elevated values in DMBC28, DMBC29 and WM266.4 cell lines. Interestingly, much higher mRNA and protein levels of RIPK4 were observed in the melanoma cells derived from lymph node metastasis (WM266.4) when compared to WM115 cells that were derived from primary melanoma of the same patient.

To preliminarily assess the significance of RIPK4 in melanoma development, we performed immunohistochemical (IHC) staining on primary and metastatic melanoma tissues, including samples from the same patients. We found that RIPK4 expression is also heterogeneously expressed in the clinical material, from low to high intensities (Figure 1c). Interestingly, we observed two different types of staining within the cytoplasm, agranular and granular, both with different intensities. A similar difference in staining was seen in xenografts obtained from melanoma cell lines with high levels of the RIPK4 kinase such as A375, WM266.4 or DMBC21 (Figure 1d). We also found that RIPK4 expression was up-regulated in xenografts when compared to original melanoma cell lines (Figure 1e). To further explore the involvement of RIPK4 in human cutaneous melanoma progression we conducted metadata analysis of RIPK4 mRNA levels in 83 clinical specimens (GDS 3966), 31 primary melanoma and 52 metastatic melanoma specimens using the Gene Expression Omnibus (GEO) database (https://www.ncbi.nlm.nih.gov/geo, accessed data 15 June 2020). This revealed similar RIPK4 mRNA levels (normalized to the log2 value) in the melanoma biopsies from primary and metastatic tumors (Figure 1g), similarly to IHC staining data in this study. While we observed no statistically significant differences between primary and metastatic melanomas, a much broader scatter of values was found within biopsies derived from metastatic melanomas than primary melanomas. Further queries of datasets for melanoma-related genetic/epigenetic changes in RIPK4 (http://www.cbioportal.org/data_sets.jsp, including the TCGA through the cBIO Portal for Cancer Genomics, accessed data 15 June 2020) demonstrated a low (6%) incidence of missense RIPK4 mutations in melanoma (Figure 1f and Supplementary Figure S1) and a reverse correlation between RIPK4 levels and methylation levels of RIPK4-encoding regions in melanoma samples (Figure 1h). Interestingly, according to cBioportal, expression at the mRNA level is associated with survival, regardless of the stage according to the Clark scale (data not shown); therefore further studies in this area are needed.

### 2.2. RIPK4 Down-Regulation Interferes with the Invasive Phenotype of WM266.4 Cells

**Figure 2:**
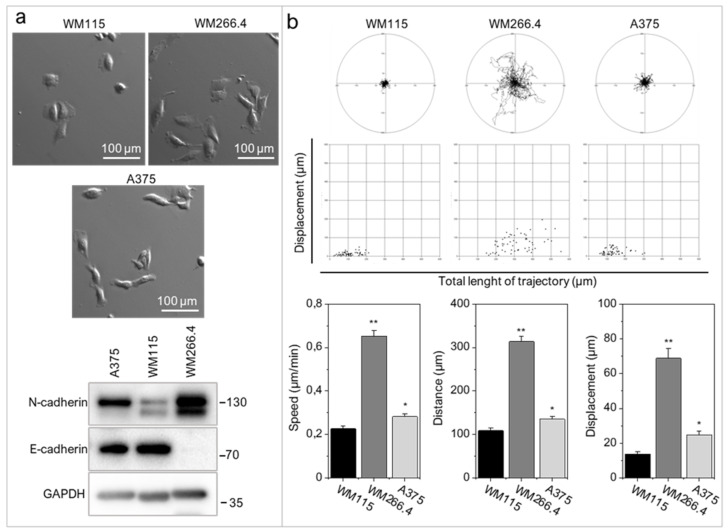
Melanoma cells with elevated levels of RIPK4 exert enhanced motile activity. (**a**) Morphology and the levels of N-cadherin and E-cadherin in WM115, WM266.4, and A375 melanoma cells. GAPDH was used as a loading control in Western blotting. (**b**) The motile activity of melanoma cells vs. WM115. Statistical analysis is based on a collection of 60 cells from three independent experiments by the Mann-Whitney Rank Sum Test * *p* < 0.05, ** *p* < 0.001.

WM266.4 cells have mesenchymal morphology (Figure 2a), considerably higher N-cadherin levels (Figure 2a) and more pronounced motile activity than WM115 and A375 cells (Figure 2b). WM266.4 cell speed was significantly higher than the speed of their primary counterparts, WM115 cells (*p* < 0.001).

**Figure 3:**
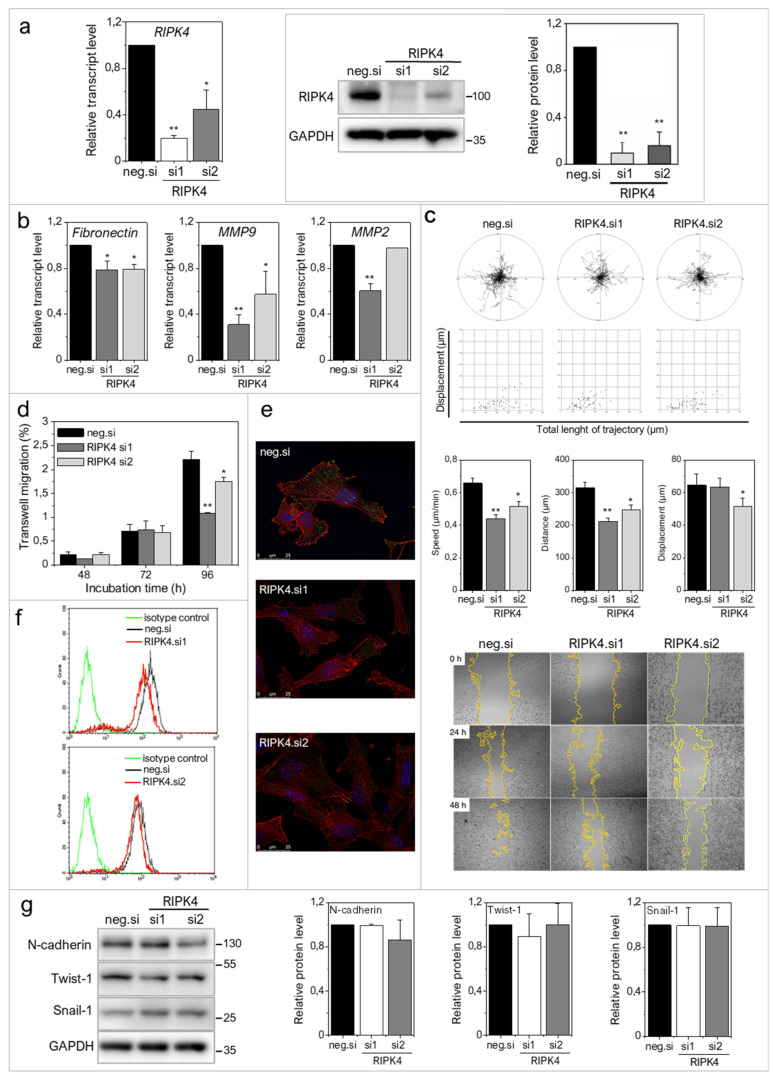
Downregulation of RIPK4 reduces WM266.4 cells motile activity. Cells were transfected with RIPK4.si1, RIPK4.si2 or neg.siRNA and induced changes were analyzed after 48 h. (**a**) RIPK4 levels in WM266.4 cells were assessed by Western blotting with densitometry and qRT-PCR normalized to GAPDH. *n* = 3. (**b**) Transcript levels of Fibronectin, MMP2 and MMP9 normalized to GAPDH. *n* = 3. (**c**) Cell motility. Statistical analysis is based on a collection of 60 cells from three independent experiments by Mann-Whitney Rank Sum Test. * *p* < 0.05, ** *p* < 0.001. Wound healing, *n* = 3. (**d**) Time-dependent migration of cells by Transwell assay. *n* = 3. (**e**) Global architecture of F-actin (red) and vinculin (green). Scale bar: 25 µm. (**f**) CD44 expression by flow cytometry. (**g**) N-cadherin and E-cadherin levels in WM266.4 cells along with densitometry. *n* = 3. All samples of RIPK4.si transfected cells were compared to neg.si (scrambled control) samples * *p* < 0.05, ** *p* < 0.001.

To address the role of RIPK4 in melanoma progression more directly, we further investigated the effects of ectopic RIPK4 down-regulation on the phenotype of highly invasive WM266.4 melanoma cells. Using RIPK4-specific small interference RNAs (siRNAs) we were able to inhibit RIPK4 levels in these cells by 70–90% (Figure 3a). Down-regulation of RIPK4 only slightly interfered with WM266.4 proliferation (Supplementary Figure S2) without causing a significant effect on apoptosis (data not shown). RIPK4 silencing resulted in decreased transcript levels of fibronectin, MMP9 and MMP2 (Figure 3b) and inhibition of WM266.4 motility (Figure 3c). Estimation of single WM226.4 cell trajectories by time-lapse video microscopy along with the results of wound healing assay demonstrated the reduced motility and displacement of melanoma cells after the ectopic RIPK4 down-regulation (Figure 3c). These data are in concordance with the results of Transwell assays (Figure 3d), which showed attenuated transmigration capacity of WM266.4 cells after RIPK4 down-regulation. The visualization of the cytoskeleton (Figure 3e, Supplementary Figure S3) revealed the F-actin rearrangements, from well-organized cytoskeletal architecture with the signs of actin polymerization at the leading edges towards thin microfilament bundles, which were weakly anchored to non-matured focal adhesions. Concomitantly, we observed a slight down-regulation of CD44, which is a receptor for hyaluronic acid (HA) involved in cell–cell interactions, cell adhesion and migration (Figure 3f). On the other hand, no significant effects of RIPK4 down-regulation on the levels of N-cadherin, Snail-1 and Twist-1 were seen (Figure 3g). These observations indicate the potential role of RIPK4 in the regulation of the invasive potential of WM266.4 cells. They also suggest the involvement of other signaling pathways in the regulation of post-EMT phenotype of melanoma cells.

### 2.3. RIPK4 Remains under the Control of Protein Kinase C-Dependent Signaling in WM266.4 Cells

**Figure 4:**
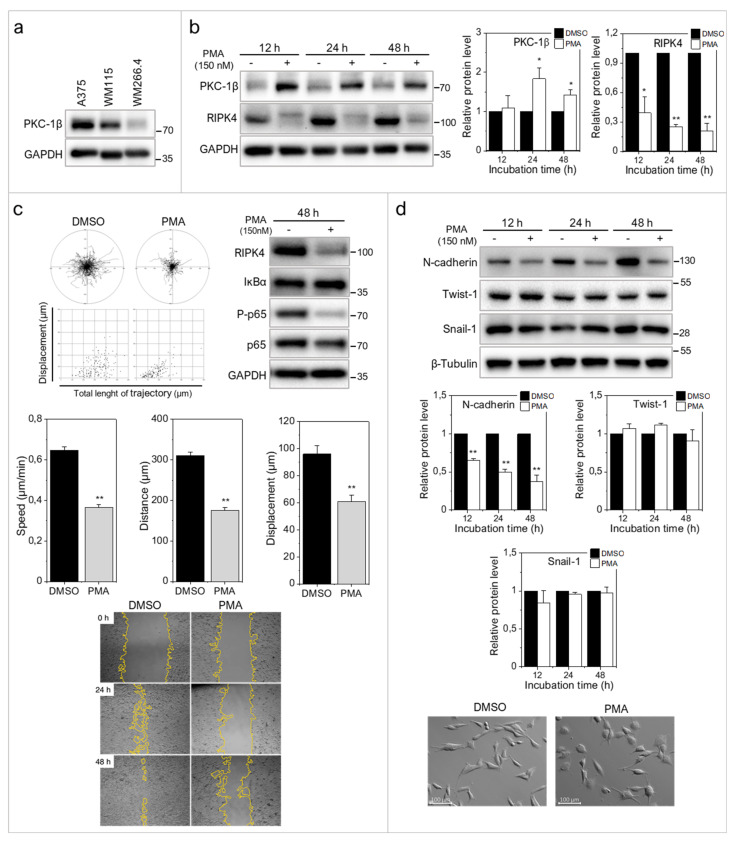
PMA decreases RIPK4 levels in WM266.4 melanoma cells. (**a**) PKC-1β level in A375, WM115 and WM266.4 cells was assessed by Western blotting. (**b**) Protein levels of PKC-1β, RIPK4 and GAPDH in WM266.4 cells treated with DMSO (control) or PMA (150 nM) for 12–48 h along with densitometry. The samples were compared to the DMSO control samples. *n* = 3 * *p* < 0.05, ** *p* < 0.001. (**c**) Motility of WM266.4 cells treated with DMSO (control) or PMA (150 nM) for 48 h. Statistical analysis is based on a collection of 150 cells from three independent experiments by the Mann-Whitney Rank Sum Test. ** *p* < 0.001. Protein levels of RIPK4, IκBα, P-p65, p65 were assessed by Western blotting. GAPDH was used as a loading control. Representative images from three independent wound healing experiments. (**d**) Protein levels of N-cadherin, Twist-1, Snail-1 and β-Tubulin along with densitometry. The samples were compared to the DMSO control samples. *n* = 3. ** *p* < 0.001. Cell morphology of cells treated with DMSO (control) or PMA (150 nM) for 48 h.

Phosphorylation of RIPK4 by PKC has previously been shown to direct RIPK4 towards proteolytic degradation [7]. To estimate the significance of PKC signaling for RIPK4 activity in WM266.4 cells and its consequences for cell motility, we treated melanoma cells with 150 nM phorbol-12-myristate-13-acetate (PMA). As expected, WM266.4 cells showed lower PKC-1β level than their WM115 counterparts (Figure 4a). PMA increased PKC1β level and reduced RIPK4 level in WM266.4 melanoma cells after 12–48 h of treatment (Figure 4b), and concomitantly inhibited the WM266.4 cell motility (Figure 4c). This reduction was even more pronounced than that caused by RIPK4 siRNA. Along with PMA-induced inhibition of the motile activity, reduced p65 phosphorylation at Ser 536, and impaired wound healing of WM266.4 cells was observed (Figure 4c). PMA treatment also induced the morphological shifts of WM266.4 cells towards non-polarized epithelioid shape and decreased the level of N-cadherin, but had no effect on Snail-1 and Twist-1 levels (Figure 4d). These analyses further confirmed the role of RIPK4 in the regulation of melanoma motile capacity in vitro and showed the functional links between RIPK4- and PKC-dependent signaling in WM266.4 cells.

### 2.4. Concerted RIPK4/PKC Signaling Regulates the Invasive Potential of A375 Cells

**Figure 5:**
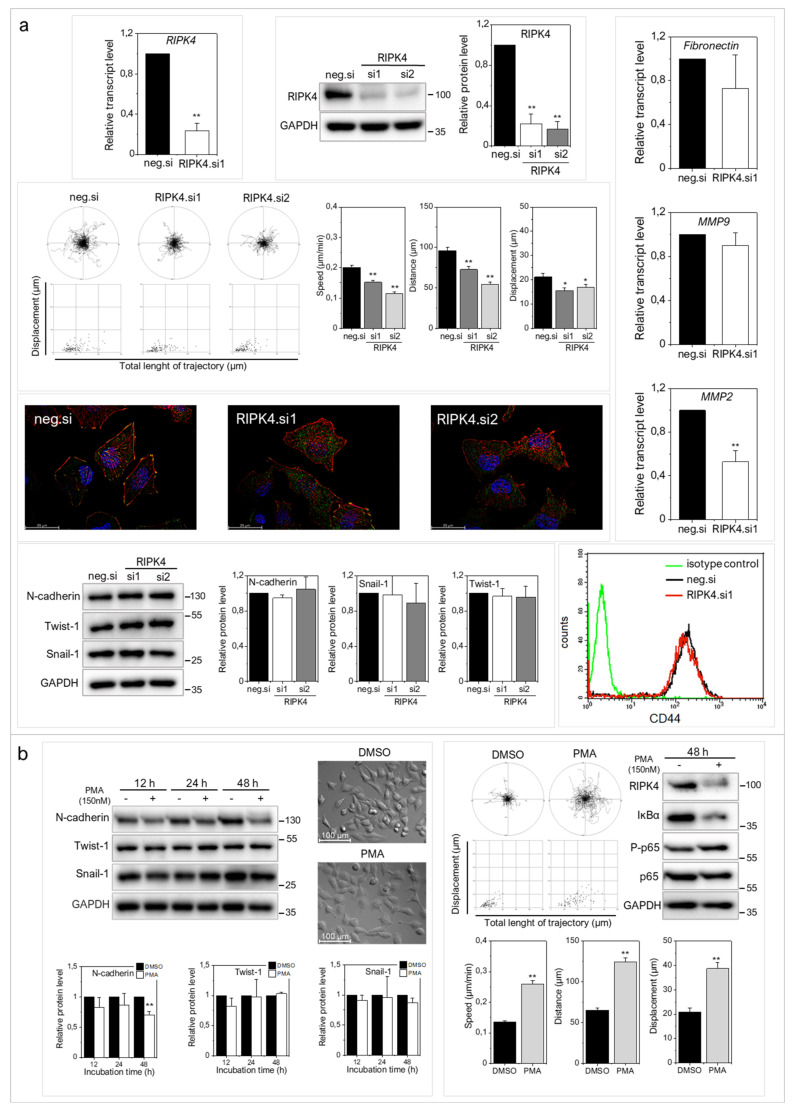
Effect of downregulation of RIPK4 and PMA the treatment on A375 cells invasive potential. (**a**) Cells were transfected with RIPK4.si1, RIPK4.si2 or neg.siRNA and induced changes were analyzed after 48 h. RIPK4 levels in A375 cells were assessed by qRT-PCR and Western blotting with densitometry normalized to GAPDH. *n* = 3. Cell motility. Statistical analysis is based on a collection of 90 cells from three independent experiments using the Mann-Whitney Rank Sum Test. * *p* < 0.05, ** *p* < 0.001. Global architecture of F-actin (red) and vinculin (green). Scale bar: 25 µm. Transcript levels of Fibronectin, MMP2 and MMP9 normalized to GAPDH. *n* = 3. Protein levels of N-cadherin, Twist-1, Snail-1 and GAPDH along with densitometry. All samples of the RIPK4.si transfected cells were compared to neg.si (scrambled control) samples, ** *p* < 0.001. CD44 expression by flow cytometry. (**b**) Cells were treated with DMSO (control) or PMA (150 nM) for 12-48 h. Protein levels of N-cadherin, Twist-1, Snail-1 and β-Tubulin along with densitometry. *n* = 3. Cell morphology and motility. Statistical analysis is based on a collection of 90 cells from three independent experiments. ** *p* < 0.001. Protein levels of RIPK4, IκBα, P-p65, p65 were assessed by Western blotting. GAPDH was used as a loading control. The samples were compared to the DMSO control samples. *n* = 3. ** *p* < 0.001.

Next, we used a combined experimental approach employing siRNA and PMA to elucidate the mechanisms underlying the influence of RIPK4 on A375 cells, in which the RIPK4 expression was originally lower (Figure 1a,b) while PKC-1β was higher (Figure 4a) than in WM266.4 cells. In contrast to WM266.4 cells, proliferation of A375 cells and cell cycle up to 72 h were not substantially affected by RIPK4 silencing (Supplementary Figure S2). Similar G1 fractions in control (51.9 ± 2.8%) and siRNA-treated variant (48.0 ± 2.9%) were seen. Similar to WM266.5 cells, A375 cells reacted to RIPK4 silencing with reduced motility (Figure 5a, Supplementary Figure S4) and MMP2 down-regulation, with no influence on the expression of Snail-1/Twist-1 was detected (Figure 5a). Furthermore, no down-regulation of MMP9, fibronectin and CD44 was observed in RIPK4 siRNA-treated A375 cells (Figure 5a). Interestingly, more pronounced differences between A375 cells and WM266.4 cells were seen after the administration of PMA for 48 h. In particular, regardless of N-cadherin decrease, no distinct morphological changes of A375 cells were observed (Figure 5b); moreover, increased motile activity was detected in PMA-treated A375 cells and accompanied by phosphorylation of p65 at Ser 536, even if PMA substantially decreased RIPK4 levels in both cell lines (Figure 4b). Collectively, these data indicate the involvement of RIPK4 in determining melanoma invasive potential, but depending on the original level of RIPK4 the extent of these effects might differ. Moreover, they also indicate that PKC activation can overcome the anti-invasive effects of RIPK4 inactivation.

### 2.5. Immediate vs. Long-Term Effect of RIPK4 Down-Regulation on NF-κB Activity in WM266.4 and A375 Cells

**Figure 6:**
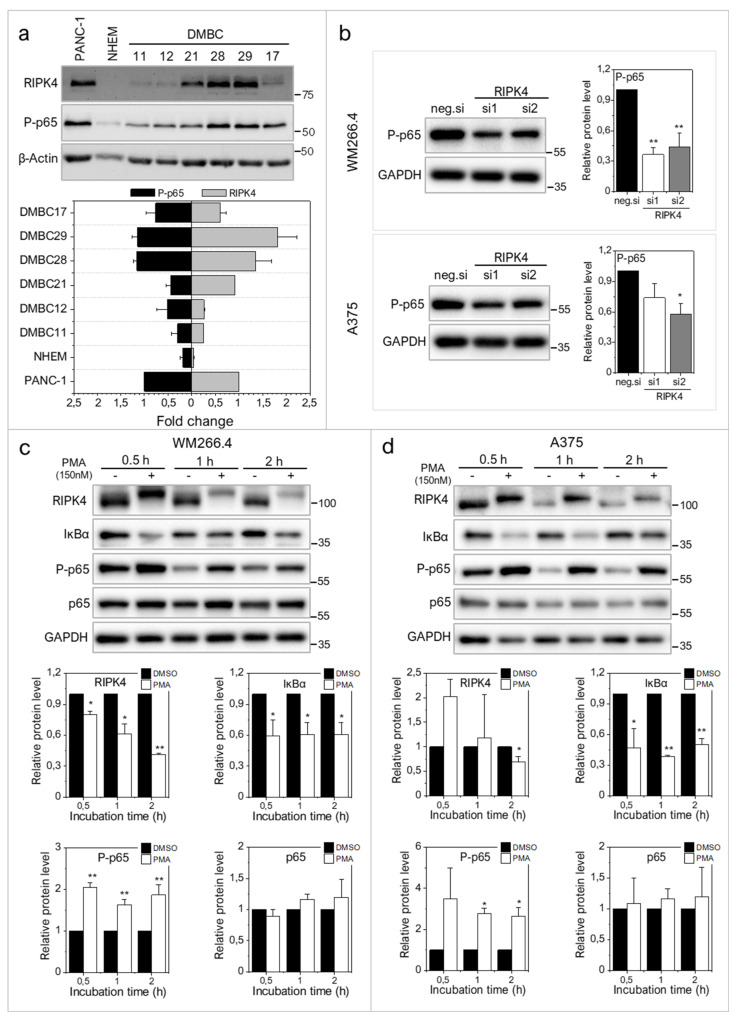
Effect of PMA short time treatment on NF-κB signaling in WM266.4 and A375 cells. (**a**) The level of phosphorylated p65 in relation to RIPK4 level in patient-derived melanoma cells with densitometry. PANC-1 cells was used as a positive control. (**b**) The level of phosphorylated p65 in cells transfected with RIPK4.si1, RIPK4.si2 or neg.si RNA by Western blotting with densitometry. The samples were compared to neg.si (scrambled control) samples. *n* = 3. * *p* < 0.05, ** *p* < 0.001. (**c**,**d**) Cells were treated with DMSO (control) or PMA (150 nM) for 0.5–2 h. (**c**) WM266.4 cell. (**d**) A375 cells. Protein levels of RIPK4, IκBα, P-p65, p65 were assessed by Western blotting. GAPDH was used as a loading control. The samples were compared to the DMSO control samples. *n* = 3. * *p* < 0.05, ** *p* < 0.001.

Long-term effects of PMA-induced down-regulation of RIPK4 depended on the cell line. In WM266.4 cells RIPK4 PMA-induced down-regulation was accompanied with reduced invasive potential and phosphorylation of p65 subunit of NF-κB, whereas in A375 cells opposite effects were detected. Before treatment, a clear positive correlation was observed between the level of RIPK4 and phosphorylation of p65 in both cell lines (Figure 4 and Figure 5) and patient-derived melanoma cell lines (Figure 6a). Downregulation of RIPK4 by siRNA decreased the level of P-p65 in WM266.4 and A375 cells (Figure 6b). As PMA influence on melanoma cells was assessed after 48 h of treatment, we asked a question about the influence of PMA after short incubation. As shown in Figure 6c,d, a short duration PMA stimulation induced phosphorylation of RIPK4 in both WM266.4 and A375 cells. This is illustrated by the presence of two RIPK4-specific bands on immunoblots (110 kDa and 97 kDa, i.e., phosphorylated and non/hypo-phosphorylated RIPK4, respectively), accompanied by the reduction of IκBα, increased phosphorylation of p65 (Figure 6b,c), and enhanced motility of WM266.4 cells (Supplementary Figure S5). These data confirm that IκBα/p65 signaling remains under RIPK4/PKC-1β control in both melanoma cell lines. Subsequent RIPK4 degradation observed in both cell lines was accompanied by the reduction of phospho-p65 levels in WM266.4 but not A375 cells after 48 h (Figure 4c and Figure 5b).

## 3. Discussion

RIPK4 has been implicated in the progression of numerous tumors [8,9,10,11,12,13,28,29]; however its role in melanoma remained unresolved. Our study fills this gap because it is the first to reveal the functional role of RIPK4 in melanoma. This role is illustrated by (i) the relatively high levels of RIPK4 in metastatic melanoma cell lineages and tumor specimens and (ii) the reduction of motility of melanoma cells by RIPK4 down-regulation. Furthermore, we show (iii) the modulatory effect of PKC that differentially influences the impact of RIPK4/NF-κB axis on the invasive potential of melanoma cell lineages. Thus, RIPK4 might play a pivotal role in the formation of a melanoma invasive front. However, in conjunction with the lack of correlation between melanoma stage and RIPK4 expression levels in ex vivo melanoma biopsies, these data also indicate the complexity of the consequences of RIPK4-dependent signaling for this process.

Abnormal cell proliferation decreased cell apoptosis and induction of cell motility play pivotal roles in skin tumorigenesis. De-Qing Liu et al. [11], demonstrated that RIPK4 knockdown significantly inhibited cell proliferation and clone formation capacity in SiHa and Caski cells. The possible role of RIPK4 in the regulation of invasive potential of melanoma was suggested by the current findings by the high RIPK4 levels in metastatic (DMBC21, DMBC28, DMBC29, WM266.4) melanoma cell lines. Further analyses of the consequences of RIPK4 down-regulation in melanoma cells revealed its clear impact on the invasive potential manifested by decreased transmigratory potential and motility of WM266.4 cells, which was also observed in A375 cells. Moreover, RIPK4-induced changes of F-actin and focal contact architecture correlated with their pro-epithelial morphological shifts, cytoskeletal rearrangements and CD44 down-regulation in WM266.4 cells. These observations correspond to the results of studies on keratinocytes, where RIPK4 participated in the maintenance of cortical F-actin organization [30,31] and intercellular junctions [32], whereas CD44 expression apparently participated to tumor invasion [33,34]. Thus, our results indicate that RIPK4 is involved in melanoma cell invasiveness by influencing cell adhesion and actin dynamics. In osteosarcomas, silencing of RIPK4 inhibited EMT by inactivating the Wnt/β-catenin signaling pathway [28]. In melanoma cells, down-regulation of RIPK4 expression by siRNA or PMA did not completely abolish the expression of EMT markers including Twist-1 and Snail-1 and N-cadherin. Thus, the lack of RIPK4 effects on Snail-1/Twist-1 levels indicates that RIPK4 exerts its pro-invasive effects downstream of EMT master regulators and seems to be related to cell adhesion.

Our mechanistic studies demonstrated that the function of RIPK4 in melanoma cells is predominantly executed through the activation of NF-κB signaling. This is illustrated by a correlation between RIPK4 and P-p65 levels in patient-derived cell lines and reduced phosphorylation of p65 in RIPK4 siRNA-treated WM266.4 and A375 cells, along with their reduced motility. The relationship between NF-κB activity and melanoma cell motility is well established [20]. RIPK4 has also been shown to promote bladder urothelial carcinoma cell aggressiveness by NFκB-induced EMT [29]. Our results also correspond to the previous reports on the down-regulation of vimentin, MMP2 and fibronectin after RIPK4 knockdown in cervical squamous carcinoma cells [11]. NF-κB has also been identified as a regulator of CD44 expression in melanocytes [33]. Thus, RIPK4/NF-κB-dependent signaling axis at least partly sustains mesenchymal phenotype of melanoma cells as additionally revealed by morphological shifts of WM266.4 cells towards an epithelioid phenotype upon RIPK4 inhibition.

Our data also show that IκBα/p65-dependent signaling remains under the control of RIPK4/PKC-1β in both melanoma cell lines. Actually, short-term PMA stimulation induced RIPK4 phosphorylation in both WM266.4 and A375 cells. This was accompanied by reduced IκBα and elevated p65 phosphorylation and induction of WM266.4 motility, additionally confirming the notion that RIPK4 activates the NF-κB signaling cascade upstream of IKKβ and IκBα [35]. These results are consistent with previous observations indicating interactions of RIPK4 with PKCβ1 and PKCδ and a role for PKC activity in the activation of NF-κB and other signaling molecules (including JNK-AP-1) [1,35]. A similar interrelation has been found in keratinocytes [36].

We also observed differences in the sensitivity of melanoma cell lines to RIPK4-dependent signaling. These are manifested by less pronounced effects of RIPK4 silencing on morphology and expression of pro-invasive markers in A375 cells than in WM266.4 cells, and by differential effects of PKC on RIPK4/NF-κB axis and the invasive potential of melanoma cell lines. Apparently, the level of phospho-p65 in WM266.4 and A375 cells after their 48 h-long PMA treatment correlated with the inhibition of WM266.4 and the induction of A375 motility. PMA reduced RIPK4 in A375 cells while retaining NF-κB activity and invasiveness of these cells. This observation indicates the direct effect of PKC on NF-κB activity and the invasive potential of A375 cells. It also shows that PKC can differentially affect NF-κB activity in discrete melanoma cell lineages. Accordingly, PKC can modulate NF-κB signaling in a RIPK4-dependent manner and/or via the RIPK4-independent pathways.

It appears that further studies are needed to fully assess the long-term consequences of RIPK4 involvement in the regulation of melanoma phenotype and microevolution of melanoma cells.

On the other hand, our data show that RIPK4 activity contributes to the invasive potential of melanoma cells. However, together with the relatively low RIPK4 levels in the biopsies of prospectively malignant melanoma tumors, they also indicate the stage-specific function of RIPK4 in melanoma development. Corresponding tumor stage-specificity has previously been documented for several other factors, including Cx43 [37,38,39]. RIPK4 exerts its inducing effect on melanoma invasiveness via NF-κB signaling; however, high susceptibility of NF-κB pathway to other signaling pathways (incl. PKC) may also determine the recruitment of RIPK4-depressed cells to the invasive melanoma front. This cell context-dependent involvement of RIPK4 in melanoma cell invasiveness, together with the suppressive function(s) of RIPK4 at the early stages of melanoma, apparently underlie the lack of straightforward correlation between RIPK4 levels and melanoma progression. Differential interplay between PKC and RIPK4 expression in discrete melanoma cells participates in this process. Further in vivo studies on clinical material from oncological patients should help to further decipher these interrelations.

## 4. Materials and Methods

### 4.1. Clinical Samples

Preliminary analysis of RIPK4 expression in human clinical samples was performed on formalin-fixed, paraffin-embedded (FFPE) samples from seven patients with diagnosed cutaneous melanoma, including samples of the primary melanomas and lymph node metastases from the same patients. The study was carried out following the rules of the Declaration of Helsinki of 1975 (revised in 2008) and the study was approved by the Institutional Review Board of Collegium Medicum, Nicolaus Copernicus University (KB136/2016) and the Bioethics Committee of the Jagiellonian University (no. 1072.6120.125.2017, date of approval 25 November 2020).

### 4.2. Geo Database Analysis

The RIPK4 expression profile deposited in Gene Expression Omnibus (GEO) (accession number GDS3966 was analyzed [40]. The microarray platforms used in these datasets were HUI133A Affymetrix DNA chips. The GDS3966 dataset comprises 83 melanoma samples (31 primary and 52 metastatic tumors) collected at the Massachusetts General Hospital and Harvard Medical School from 1992 to 2001 as a part of the diagnostic workup or planning for therapy. As claimed in the original papers, all the studies had been approved by the local ethical committees and all participants gave written informed consent [40]. The gene expression level was normalized to the log2 value.

### 4.3. Xenografts

The experiments were carried out according to the guidelines of the I Local Ethics Committee of the Institute of Pharmacology of the Polish Academy of Sciences (approval no. 411/2020 and 461/2020, date of approval 24 June 2020, 25 November 2020). The mice were handled according to the regulations of the national and local animal welfare bodies under SPF (Specific-pathogen-free) conditions, with sufficient water and food provided at all times. The 4- to 6-week old female NOD-SCID mice (non-obese diabetes severe combined immunodeficiency mice) were injected subcutaneously with 3 × 10^6^ A375, WM266.4 and DMBC21 cells/mouse. Experiments were carried out with three NOD-SCID mice/group. Mice were subjected to anesthesia and xenografts were harvested after 36 days (A375 cells), 41 days (WM266.4 cells), and 55 days (DMBC21). Tumor samples were divided into two pieces, one group was fixed in 10% formalin and the second was frozen at −80 °C for RNA isolation.

### 4.4. Cell Culture and Treatment

Normal, human, adult melanocytes were obtained from Lonza in 2020 (Lonza, Basel, Switzerland). Melanocytes were cultured in M254 medium supplemented with human melanocytes growth supplement (HMGS, Invitrogen, Thermo Fisher Scientific, Waltham, MA, USA). Human melanoma cell lines: Wistar Melanoma collection cells: WM115 (RRID:CVCL_0040; primary melanoma, VGP—vertical growth phase) and WM266.4 (RRID:CVCL_2765; metastatic human melanoma cell line, derived from the same patient as WM115) kindly provided by Dept. of Medical Biochemistry, Jagiellonian University Medical College (Kraków, Poland) in 2006 and have been authenticated using STR profiling. A375 cells (RRID:CVCL_0132; metastatic human melanoma cell line) were obtained from ATCC (Manassas, VA, USA) in 2020. Melanoma cells were cultured in RPMI1640 medium supplemented with 10% FBS (Gibco, Paisley, UK) and antibiotics (penicillin 150  U/mL, streptomycin 100 µg/mL, Sigma-Aldrich, Saint Louis, MO, USA) at 37 °C at 5% CO_2_ and 95% humidity. DMBC cell lines (Department of Molecular Biology of Cancer, DMBC) were obtained from drug-naïve melanoma patients during surgical interventions. The study was approved by the Ethical Commission of Medical University of Lodz (RNN/84/09/KE) and informed consent was obtained from all patients. Cells were culture in condition described previously [41]. Whole-Exome Sequencing raw data of DMBC cell lines are publicly available under the accession numbers E-MTAB-6978. Data were mapped to the reference genome GRCh37/hg19 using BWA package (version bwa-0.7.12) [41]. Phenotypes of these cells were also extensively characterized [42]. PMA (150 nM, Sigma-Aldrich, Saint Louis, MO, USA) was dissolved in DMSO and added to cells culture for 0.5–48 h. DMSO concentration does not exceed 0.05%. All experiments were performed with mycoplasma-free cells (Lonza, Basel, Switzerland).

### 4.5. Immunostaining

4µm FFPE sections, after deparaffinization, rehydration, and heat-induced antigen retrieval using low pH buffer (Vector Laboratories, Inc., Burlingame, CA, USA) and quenching the endogenous peroxidase with 3% H_2_O_2_, were incubated with primary anti-RIPK4 antibody (recognizing recombinant fusion protein containing a sequence corresponding to amino acids 240–520 of human RIPK4; ABclonal, Woburn, MA, USA) overnight at 4 °C. Next, the HRP-conjugated secondary antibody (ImmPRESS HRP REAGENT KIT anti-Rabbit IgG, Vector Laboratories, Inc., Burlingame, CA, USA) followed by ImmPACT NovaRED (Vector Laboratories Inc., Burlingame, CA, USA) and counterstaining with hematoxylin were applied. Duodenum served as positive control. In negative controls, the primary antibodies were omitted and replaced with antibody diluent. The sections were evaluated semi-quantitatively, as previously described [43,44]. Since we observed heterogenous: agranular and granular staining, the assessment was performed separately for each staining pattern.

### 4.6. Cell Transfection

For RIPK4 downregulation two types of small interfering RNA (siRNA) were used: RIPK4-specific Silencer Select siRNAs (ID: s28865 and s28863, Thermo Fisher Scientific, Waltham, MA, USA) or Silencer Select Negative Control No. 2 (cat.no 4390846, Thermo Fisher Scientific, Waltham, MA, USA), serving as a negative control. The transfection was performed, as previously described [45]. The levels of RIPK4 in siRNA transfected cells was verified by Western blot or qRT-PCR analysis.

### 4.7. Western Blot

Western blot analysis was carried out as described previously [46], except for lysates from DMBC lineage cells which were analyzed as described [41]. The blots were cut prior to hybridization with antibodies. Labelled bands were detected with ChemiDoc Imaging System (BioRad, Hercules, CA, USA). Densitometry analysis was performed using ImageJ software or ImageLab 5.2.1. software. We used the following antibodies and dilutions: rabbit IgG anti-human: RIPK4, N-cadherin, P-p65 (Ser-536), p65, GAPDH, IκBα (1:2000, Cell Signaling Technology, Danvers, MA, USA), PKC1β (1:2000, Invitrogen, Thermo Fisher Scientific, Waltham, MA, USA), E-cadherin, Twist-1, Snail-1 (1:2000, Sigma-Aldrich, Saint Louis, MO, USA), β-Tubulin (1:2000, Abcam, Cambridge, UK), β-Actin (1:4000, Santa Cruz Biotechnology, Dallas, CA, USA), goat HRP-conjugated anti-rabbit (1:4000, Cell Signaling Technology Danvers, MA, USA) and goat HRP-conjugated anti-mouse (1:4000, BP Pharmingen, NJ, USA).

### 4.8. RNA Isolation and Quantitative RT-PCR

Total RNA was isolated using the Total RNA Mini Plus (A&A Biotechnology, Gdansk Poland) accordingly to the manufacturer’s protocol. RNA (1µg) was reverse-transcribed into cDNA using oligo(dT)18 and TranScriba Kit (A&A Biotechnology, Gdansk Poland) or using SuperScript II Reverse Transcriptase (Invitrogen, Thermo Fisher Scientific, Waltham, MA, USA). qRT-PCR reactions were performed using Sensitive RT HS-PCR Mix (A&A Biotechnology, Gdansk, Poland) and the qTOWER^3^ real-time PCR thermal cycler (Analytik Jena, Jena, Germany) or the KAPA SYBR FAST qPCR and Rotor-Gene 3000 Real-Time DNA analysis system (Corbett Research, Mortlake, Australia). Primer sequences were as follows: RIPK4 forward: 5′-ATG CCC ACT ACC ACG TCA AG-3′ and reverse 5′-TCT TCT CAT CTG CAA ACG GCT-3′; RPS17 forward 5′-AAT CTC CTG ATC CAA GGC TG-3′ and reverse 5′-CAA GAT AGC AGG TTA TGT CAC G-3′. A mathematical model including an efficiency correction was used to calculate relative expression of selected genes versus a reference gene RPS17. All TaqMan primers were from Thermo Fisher Scientific/Invitrogen: RIPK4 (Hs01062501_m1), Fibronectin-1 (Hs01549976_m1), MMP2 (Hs01548727_m1), MMP9 (Hs00957562_m1), GAPDH (cat. No. 4326317E). The relative levels of transcripts were quantified by the 2^−ΔΔCt^ method, using GAPDH as a reference gene.

### 4.9. Immunofluorescence

2 × 10^4^ cells were seeded on coverslips in a 12-well plate and incubated for 24–96 h. For immunolocalization of cytoskeletal proteins (vinculin, F-actin) cells were fixed and labeled as described previously [47] and stained with primary antibodies rabbit anti-vinculin (1:200 Sigma-Aldrich, Saint Louis, MO, USA), secondary antibodies AlexaFluor488-conjugated chicken anti-rabbit and TRITC-conjugated phalloidin (1:50). For DNA visualization the cells were stained with Hoechst 33258 (Sigma-Aldrich, Saint Louis, MO, USA). The specimens were visualized by TIRF imaging using a DMI6000B microscope.

### 4.10. Time-Lapse Video Microscopy

Analysis of cells’ motility was performed using time-lapse video microscopy. Cells were seeded in a 24-well plate and subjected to microscopic analysis 40 h after PMA or siRNA treatment, within environmental control chamber custom adjusted to the procedure. Images were registered every 5 min within 8 h span. Images were acquired using fully automatic DMI6000B (Leica AF7000 version, Wetzlar, Germany) and analyzed with Hiro program [47].

### 4.11. Transmigration Tests

15 × 10^3^ cells were seeded on the upper side of trans-well migration inserts (Corning, NJ, USA) in a 24-well plate and allowed to transmigrate in hemodynamic conditions for 24 h. Afterward, the inserts were transferred to another well for 24 h and the procedure was repeated 4 times. Cells in the wells were counted to determine the initial number of transmigrating melanoma cells [48].

### 4.12. Wound Healing Assay

The scratch wound was made using 200 μL sterile pipette tip in a confluent cell culture pre-treated with DMSO, PMA or transfected with negative siRNA (scrambled control) or RIPK4.siRNAs The scratch area was washed, and cells were re-incubated in the same condition for additional 48 h. The images were taken at 0, 24 and 48 h time points. The ImageJ software was used to draw the lines of the healing wound areas.

### 4.13. Flow Cytometry Analysis of CD44

Cells were washed with PBS containing 0.1 mM EDTA and counted with a Bürker hemocytometer. Then 5 × 10^5^/^mL^ cells were incubated for 30 min on ice in PBS/EDTA supplemented with 1% BSA and rat anti-CD44-FITC conjugated antibodies (clone IM7, 1:600 Thermo Fisher, Waltham, MA, USA) or its isotype match control (Rat IgG2bκ Isotype Control) and washed twice. Afterward, viable cells were gated based on FSC/SSC scatter and 10,000 cells were collected by FACS Calibur instrument (BD Biosciences, San Jose, CA, USA) and analyzed with CellQuest (BD Biosciences) software. The measurement was carried out using 488 nm excitation and a 510–570 nm band-pass emission filter for the detection of fluorescein isothiocyanate (FITC).

### 4.14. Cell Cycle

Cells were trypsinized and washed with PBS followed by fixation with ice-cold EtOH (70%) and stored at −20 °C. Cells were washed and stained as described [49]. Viable cells were gated based on FSC/SSC scatter and collected by FACS Calibur instrument (BD Biosciences, San Jose, CA, USA). Data were analyzed using the ModFit LT 5 software.

### 4.15. Statistical Analysis

Graphs are presented as a mean of at least three independent experiments ± SD unless stated otherwise. To compare two independent samples, the Student’s *t*-test was used. The differences between primary and metastatic melanomas were measured Student’s *t*-test with Wilcoxon matched-pairs signed ranked for paired data from the same patients. Differences were considered significant at *p* < 0.05.

## 5. Conclusions

Our data show that RIPK4 contributes to melanoma development. Apparently, this effect is achieved in a manner dependent on NF-kB signaling. A selective recruitment of RIPK4^low^ and RIPK4^high^ discrete melanoma cell lineages to the invasive front of melanoma is determined by a differential sensitivity of NF-κB signaling to alternative (PKC-related) activators. Our data also confirm that PKC-dependent signaling can affect the invasive potential of discrete melanoma cell lineages in a manner dependent on the duration of PKC activation. Therefore, the interaction between the RIPK4, PKC and NF-κB pathways [25,27] is involved in the progression of melanoma [50].

## Data Availability

Data sharing not applicable. No new data were created or analyzed in this study. Data sharing is not applicable to this article.

## Supplementary Materials

The following are available online at https://www.mdpi.com/article/10.3390/ijms222111504/s1.
