# Supplementary material for: Deciphering the Functional Role of RIPK4 in Melanoma
Source: Int J Mol Sci. 2021 Oct 25;22(21):11504. doi: 10.3390/ijms222111504 (PMC8583870; doi:10.3390/ijms222111504)
Supplement: Supplementary file 1 [file ijms-22-11504-s001.zip › ijms-1412884-supplementary.pdf]

Deciphering the functional role of RIPK4 in melanoma

Ewelina Madej[1], Damian Ryszawy†[2], Anna A. Brozyna[3], Jaroslaw Czyz[2] Malgorzata Czyz[4] and Agnieszka Wolnicka-Glubisz [1,*]

[1] Department of Biophysics, Faculty of Biochemistry, Biophysics and Biotechnology, Jagiellonian University, Kraków, Poland; e-mail: ewel.madej@doctoral.uj.edu.pl; a.wolnicka-glubisz@uj.edu.pl

[2] Department of Cell Biology, Faculty of Biochemistry, Biophysics and Biotechnology, Jagiellonian University, Kraków, Poland; e-mail: jarek.czyz@uj.edu.pl

[3] Department of Human Biology, Faculty of Biology and Veterinary Sciences, Nicolaus Copernicus University, Toruń, Poland; e-mail: anna.brozyna@umk.pl

[4] Department of Molecular Biology of Cancer, Medical University of Lodz, Lodz, Poland; e-mail: malgorzata.czyz@umed.lodz.pl

† Damian Ryszawy, PhD passed away on 11.09.2020

*Correspondence: ul. Gronostajowa 7, 30-387 Kraków, Poland; E-mail: a.wolnicka-glubisz@uj.edu.pl, Tel.: (+48)126646526

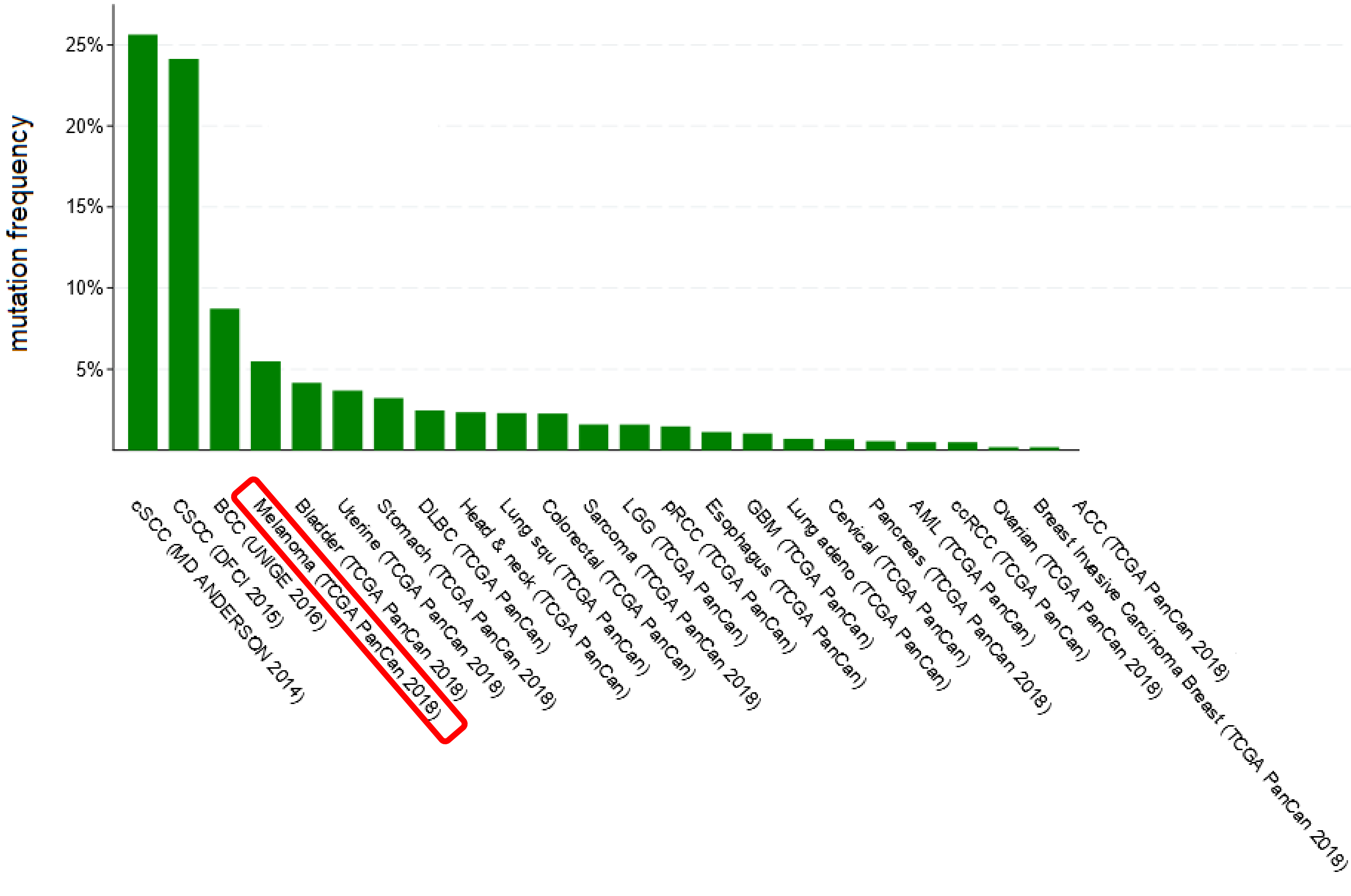

FIGURE S1 Frequency of mutations in RIPK4 in human melanoma obtained from cBIOPortal. The Cancer Genome Atlas (TCGA) was queried for mutations in the *RIPK4* gene. Shown are the frequencies of mutations for all datasets, out of 447, in which mutations in RIPK4 were identified

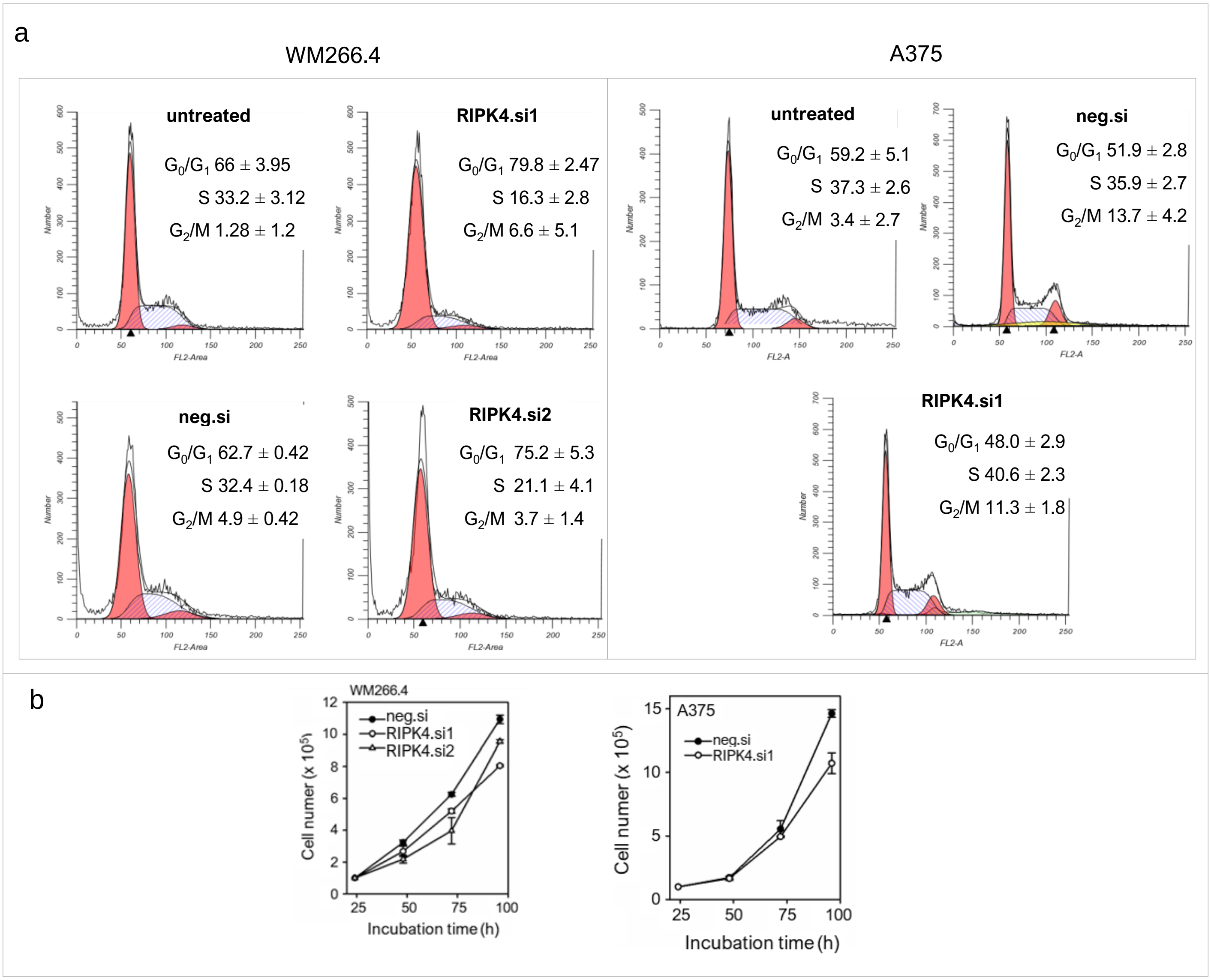

FIGURE S2 Effect of siRNA down-regulation of RIPK4 on cells proliferation. (**a**), Cell cycle profiles 72 h after transfection on WM266.4 and A375. (**b**) cell proliferation by cell counting

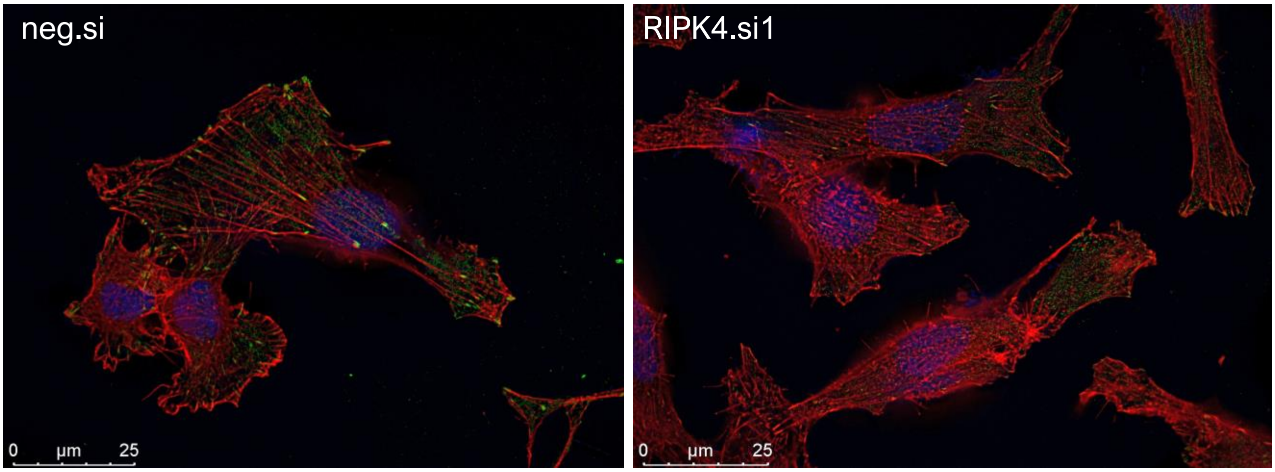

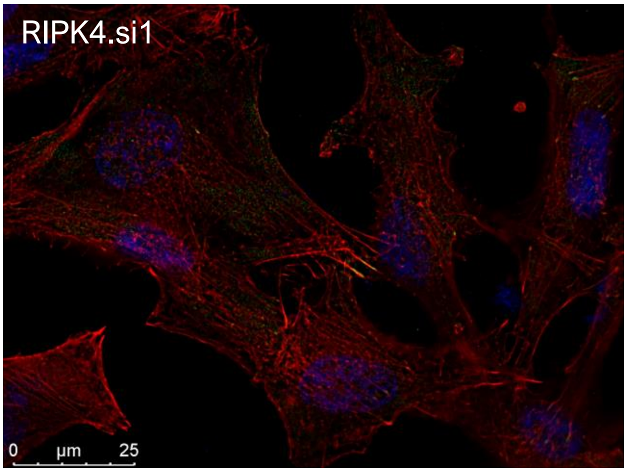

FIGURE S3 Architecture of F-actin and vinculin 48 h after transfection WM266.4 cells with neg.si (scrambled control, upper left), RIPK4.si1 (upper right), and RIPK4.si2 (lower left)

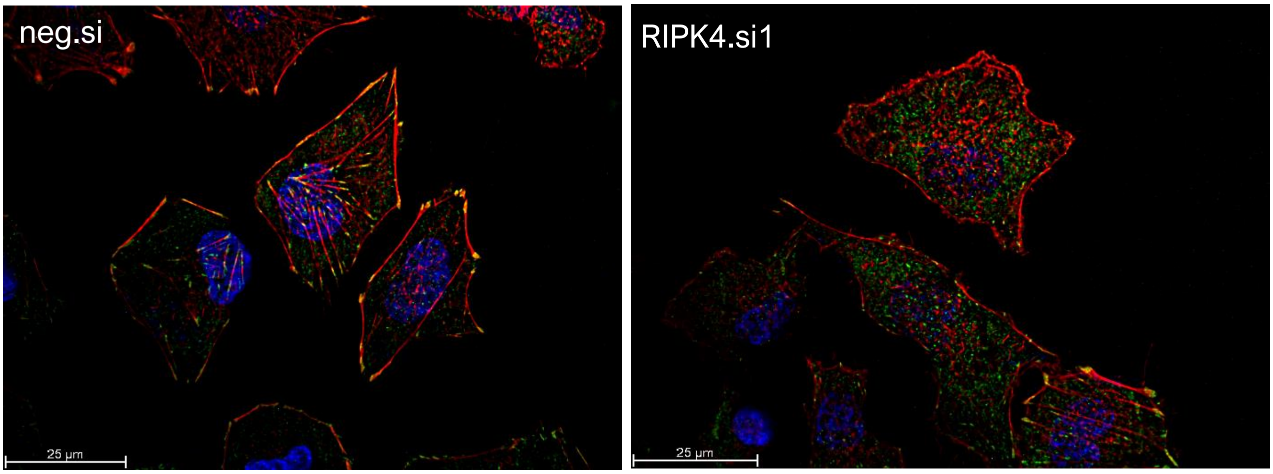

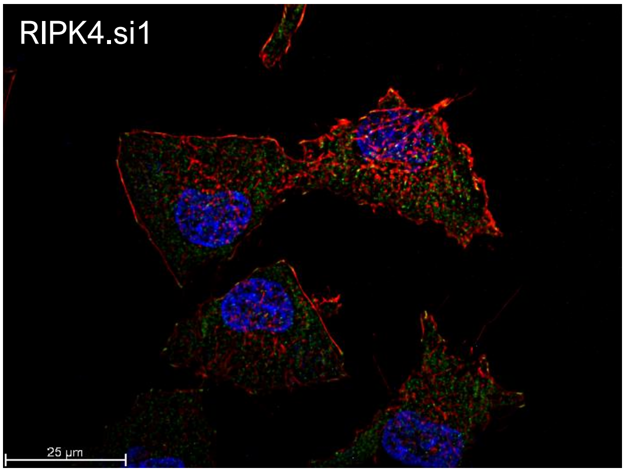

FIGURE S4 Architecture of F-actin and vinculin 48 h after transfection A375 cells with neg.si (scrambled control, upper left), RIPK4.si1 (upper right), and RIPK4.si2 (lower left)

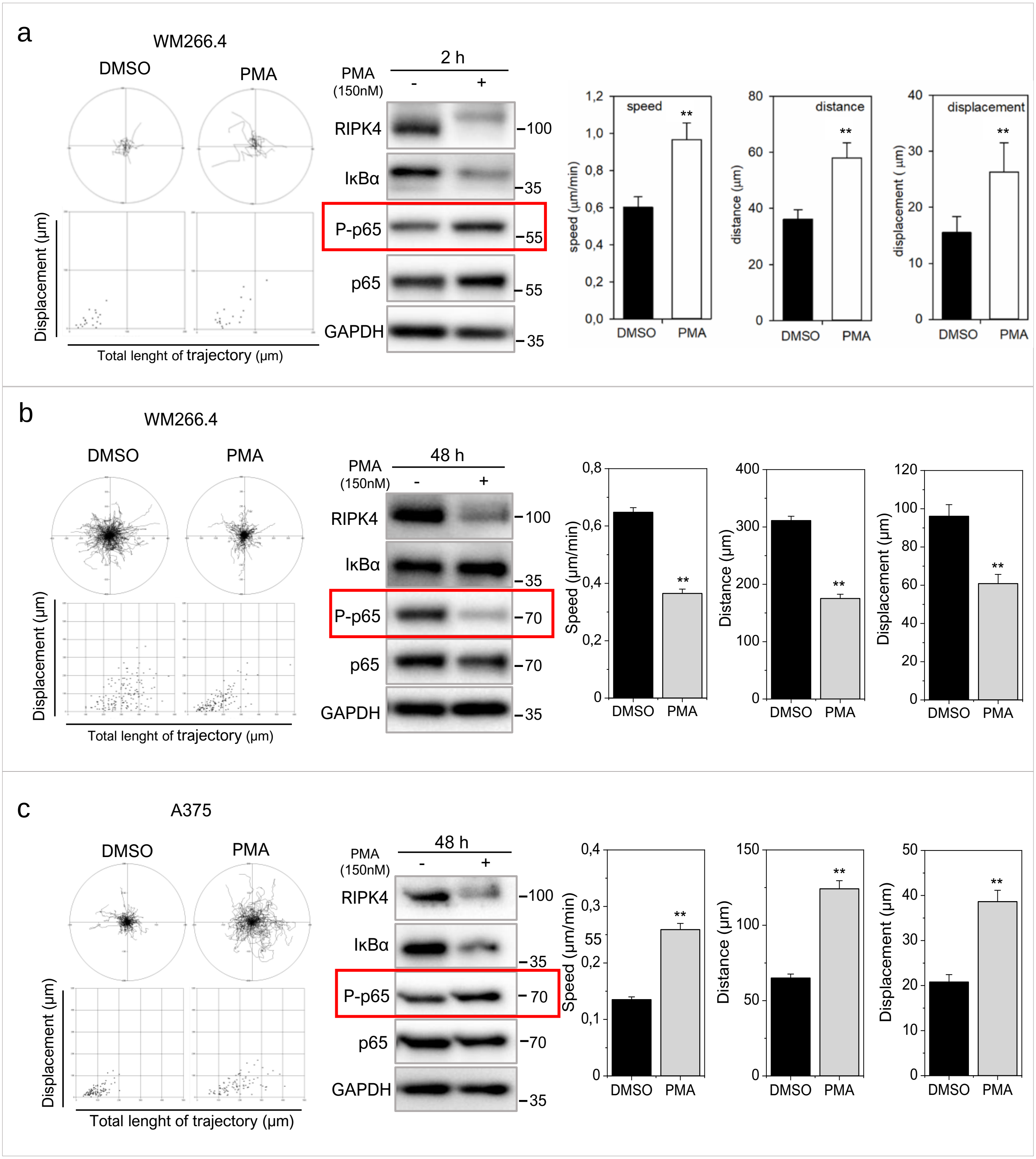

FIGURE S5. Motile activity corelates with p-p65 levels in melanoma cells after PMA treatment. Cells were treated with PMA or DMSO for (**a**) 2 h or (**b**,**c**) 48 h. ** *P < 0.001*
